# Previous contraceptive treatment relates to grey matter volumes in the hippocampus and basal ganglia

**DOI:** 10.1038/s41598-019-47446-4

**Published:** 2019-07-29

**Authors:** Belinda Pletzer, TiAnni Harris, Esmeralda Hidalgo-Lopez

**Affiliations:** Department of Psychology & Centre for Cognitive Neuroscience, University of Salzburg, Salzburg, Austria

**Keywords:** Cognitive neuroscience, Neurogenesis

## Abstract

Oral contraceptive (OC) effects on the brain have gained increasing interest, but are highly controversial. Previous studies suggest that OC users have larger hippocampi, parahippocampi, fusiform gyri and Cerebelli. Preliminary evidence from one of those studies even suggests an effect of previous contraceptive use on the hippocampi of women who are not current users of OCs. Furthermore, more recent studies postulate an involvement of previous OC treatment in later development of mood disorders. To address the question whether previous OC treatment affects women’s brain structure later in life, high resolution structural images were obtained from 131 naturally cycling women. Among them, 52 women had never used OC before, 52 had previously used one OC for a continuous time period and 27 had previously used multiple contraceptives. The groups did not differ in gray matter volumes. Since endogenous sex hormones modulate gray matter volumes of the hippocampus and basal ganglia along the menstrual cycle, we hypothesize effects of OC use on these areas. Specifically, we hypothesize that a longer duration of previous OC treatment is related to larger hippocampi and larger basal ganglia. Indeed we found the duration of previous OC use to be positively correlated to hippocampal and basal ganglia volumes bilaterally. For the hippocampus, but not for the basal ganglia, this association disappeared after controlling for the time since discontinuation. These results suggest that for the hippocampus, but not for the basal ganglia, effects of previous contraceptive treatment are reversed after a time period comparable to treatment duration. These data question the immediate reversibility of OC effects on brain structure. Accordingly, some changes in the brain due to long-term contraceptive use, while subtle, may be long-lasting.

## Introduction

In developed countries about half of the women at childbearing age rely on hormonal contraception and almost every woman has used hormonal contraception at some point in her life^1^. The most common hormonal contraceptives are combined oral contraceptives (OC), typically containing 15–40 µg ethinylestradiol (EE) and varying levels of some synthetic progestin. Ethinylestradiol is a very potent synthetic estrogen with a longer half-life and higher estrogen receptor binding-affinity compared to the endogenous estrogen estradiol^2^. Progestins are a heterogenous class of synthetic steroids exerting progestogenic actions. They differ in their binding-affinity to a variety of steroid-receptors such as the progesterone-receptor, the androgen-receptor, the glucocorticoid-receptor and the mineralocorticoid-receptor^3^, with varying clinical properties as a result. Older-generation progestins are derivates of 19-nortestosterone, a common anabolic steroid^4^. These side-effects have often been attributed to their androgenic actions^3,5^. The most common second generation progestin is *Levonorgestrel*, for which androgenic side-effects have previously been described, even in combination with EE^6^. Levonorgestrel is still widely used, not only in combined OCs, but also in subdermal implants and intra-uterine devices. Third generation progestins, like *Desogestrel*, *Norgestimat*, *Gestoden* and *Dienogest*, comprise a mix of androgenic and anti-androgenic progestins^6^, but their use has decreased over the past years. Newer progestins (fourth generation) on the other hand, bind with higher specificity to the progesterone- and mineralocorticoid-receptor and are anti-androgenic^5^. Particulary *Drospirenone*, derived from Spirolactone, is commonly used in newer OCs. However, also older progestins, derived from 17-hydroxyprogesterone are anti-androgenic, including e.g. *Chlormadinone acetate* and *Cyproterone acetate*, which are seldomly used in modern OC.

About 150 million women worldwide use OC for increasingly longer periods of time^7^. Accordingly, OC have been studied extensively with the goal to reduce the adverse side effects on physical health and emotions^8^. However, the effects of OC on brain structure and function have hardly been investigated^9^, even though accumulating evidence suggests an impact of endogenous sex hormones on the female brain, particularly the hippocampus^10–14^. It has consistently been demonstrated that hippocampal volumes increase with rising estradiol levels in the pre-ovulatory cycle phase^10–13^. Also hippocampal activation across two different cognitive tasks shows an estradiol-related increase during the pre-ovulatory cycle phase^14^. Furthermore, studies on endogenous sex hormones suggest a progesterone-dependent increase in right basal ganglia volumes and an increase in right fronto-striatal activation during the luteal cycle phase of the menstrual cycle^12–14^. Accordingly, these structures are also likely targets for the actions of synthetic steroids. Since endogenous progesterone seems to be opposing the effects of endogenous estradiol on the hippocampus^10–13^, and OC contain both an estrogenic and progestogenic component, it is hard to make a specific prediction regarding their effects on the hippocampus. However, given the high relative potency of ethinylestradiol, we still expect estrogenic effects of OCs in the hippocampus. In accordance with this assumption, users of estrogen replacement therapy during menopause show increased hippocampal volumes^15,16^. Likewise, progestogenic effects of OCs can be expected in the right basal ganglia, and probably also the right frontal cortex.

However, brain structural changes in response to OC intake have not been evaluated in a within-subjects design and only few studies have looked at differences in brain structure between OC users and non-users^17–22^. These studies suggest larger regional gray matter volumes in the hippocampus, parahippocampus, fusiform gyri and Cerebellum in OC users compared to naturally cycling women^17–19^. Differences in the basal ganglia have so far only been reported by one study^17^. Furthermore, differences between OC users and non-users have been reported in the prefrontal cortex and amygdala^17–22^. However, these findings are inconsistent. While some studies report larger volumes in pre-frontal areas for OC-users^17^, other studies report smaller volumes in pre-frontal areas for OC-users^19,20^. It has been discussed that the androgenicity of the progestin component may play a role in that respect^9,19^. In the study that found larger frontal cortex volumes in OC-users^17^, most participants were likely users of anti-androgenic progestins [compare^19^]. A reduction in frontal cortex volumes has however been observed in users of androgenic OCs^19,20^. Furthermore, OC effects on the fusiform gyrus were reported for users of anti-androgenic OCs, but not for users of androgenic OC^17,19^. However, in a recent study, smaller hippocampal and fusiform volumes were observed in OC users compared to non-users^23^. Note however, that this likely comprised a mixed group of OC users, since most women in this study were unable to provide details on the OC they were using. These inconsistencies between studies show, that group comparisons between OC-users and non-users are problematic, since they are prone to sampling bias. OC users and non-users likely differ in relationship status, probably in social status and – most importantly – the fact whether they tolerate OC without experiencing side effects. Unfortunately, longitudinal studies confirming the results obtained by group comparisons are still lacking.

Nevertheless it has been discussed, whether the effects of OC are fully reversible or whether some of the OC-dependent changes persist beyond the period of contraceptive treatment. Recent studies have implicated previous contraceptive treatment in the development of mood disorders like major depression^24–26^. Accordingly, the question arises, if some brain areas still show effects of previous contraceptive use later in life. Menstrual cycle dependent changes, e.g. in the hippocampus^9–14^ suggest a quick remodelling of brain structure by endogenous sex hormones. If the effects of synthetic steroids contained in OC follow the same mechanism, full reversibility of OC effects is to be expected. However, menstrual cycle dependent changes have been reported to be rather small^13^ and follow only short and restricted periods of exposure to increased levels of estradiol and progesterone for about a day (pre-ovulatory estradiol peak) or a week (mid-luteal progesterone peak). On the contrary, synthetic steroids like ethinylestradiol have a higher potency than endogenous steroids^2^ and female brains are exposed to those steroids for substantially longer periods than during a natural menstrual cycle. Exposure persists for at least three weeks within one treatment cycle (active pill phase) and may persist for several years, even decades, over the whole treatment period. Preliminary data from a small subsample in our previous study suggest, that even in women, who are not current users of OC, the duration of previous contraceptive use affects absolute hippocampal volumes^19^. The longer women had previously used OCs the larger were their grey matter volumes in the hippocampus bilaterally. This is in line with the assumption of estrogenic actions of ethinyestradiol on the hippocampus. However, also women with higher testosterone levels demonstrate larger hippocampi^23^, such that androgenic actions of previous OCs may also play a role in the reported effect. However, for effects of endogenous testosterone, it is unclear whether they are mediated via androgen receptors or estrogen receptors, since testosterone can locally be quickly metabolized to estradiol via the enzyme aromatase. Androgenic progestins on the other hand have no estrogenic effects, but act via the androgen receptor. Conversely, anti-androgenic progestins have been shown to also exert estrogenic actions. Accordingly, anti-androgenic progestins may enhance the actions of ethinylestradiol, while androgenic progestins may exert additional effects in the hippocampus via androgen receptors.

Thus, even though the brain appears to be very plastic in response to small, short-lasting cyclic changes in naturally cycling women, the question arises whether long-term exposure to synthetic steroids may result in more pronounced changes that are not fully or only slowly reversed after termination of contraceptive treatment. In the present study we follow up on this question using a large sample of 131 naturally cycling women. One problem that is to be expected when addressing previous contraceptive use, is that not all women are able to recall, which brand they had used several years ago. Thus, reliably addressing the modulatory role of androgenicity is hard. Furthermore, many women will have used different types of OCs, which makes it hard to disentangle the contributions of androgenic and anti-androgenic OCs. To deal with this problem, we focus our analysis on a subsample of 52 women, who report previous use of only one OC for a continuous time period. Furthermore, duration of previous contraceptive treatment is likely dependent on the age of participants^19^, which also relates to brain volumes. To avoid such collinearities and compare different treatment durations in women of comparable age, we restrict the age range of participants to 18–35 years. Finally, we focus our analysis on those brain areas, for which effects can – in theory – be expected irrespective of androgenicity, i.e. the hippocampus and the basal ganglia. Specifically, we hypothesize that women with a longer duration of previous contraceptive treatment have larger hippocampal volumes. As outlined above, it is unclear whether the androgenicity of the progestin component plays a role in that respect, but the primary effect is assumed to depend on estrogenic actions by ethinylestradiol and should thus be present for both types of OCs. Likewise, we hypothesize that women with a longer duration of previous contraceptive treatment have larger volumes of the basal ganglia due to progestogenic actions of synthetic progestins. Again, both types of progestins act on progesterone receptors and the effect should thus be present in both types of progestins. Nevertheless, for both hypotheses, moderating effects of androgenicity will be explored. We furthermore explore, whether any other brain areas outside the hippocampus or basal ganglia are affected by previous contraceptive use and whether the time since discontinuation relates negatively to hippocampal and basal ganglia volumes.

## Results

Among the 131 women tested, 52 had never used any hormonal contraceptives, 52 had used only one OC before for a continuous time period of at least 3 month, and 27 had used multiple different contraceptive methods. Since the respective influence of various contraceptive methods is hard to disentangle, we focus our analysis on the 52 women, who had used only one OC before. Never users and users of one previous OC did not differ in IQ, education levels or menstrual cycle duration (all t <1.31, all p > 0.19), but participants who had never used OCs were significantly younger (t_(102)_ = −2.25, p = 0.03). Total intracranial volume, total GM and total WM did not differ between never users and users of one OC (all t <1.90, all p > 0.06) and neither did GM volumes in the bilateral hippocampus and bilateral basal ganglia did after controlling for age and TIV (all F <0.95, all p > 0.33). Descriptives are summarized in Table 1.

**Table 1:** Age, cycle duration and brain volumes of women who had never used oral contraceptives (OC), women who had used only one OC before and women who had used multiple contraceptive methids before.

	never OC (n = 52)		one OC (n = 52)		Multiple OC (n = 27)	
	mean	SD	mean	SD	mean	SD
age	22.73	3.31	24.40	4.21	26.70	3.63
Cycle duration	28.19	2.68	28.91	2.90	28.59	2.56
TIV	1451.00	94.77	1424.00	95.39	1465.00	120.40
GM	702.80	46.02	685.60	46.03	695.50	56.28
WM	482.20	42.30	471.90	35.65	491.80	55.83
HippocampusL	4.25	0.29	4.23	0.45	4.24	0.45
HippocampusR	3.84	0.25	3.84	0.35	3.93	0.32
BG_L	9.18	0.85	9.08	0.74	8.94	1.11
BG_R	9.57	0.88	9.51	0.76	9.22	1.05

SD = standard deviation. TIV = total intracranial volume, GM = gray matter, WM = white matter, BG = basal ganglia, L = left, R = right.

### Relationship of previous OC treatment to grey matter volumes

#### Hippocampus

**Figure 1:**
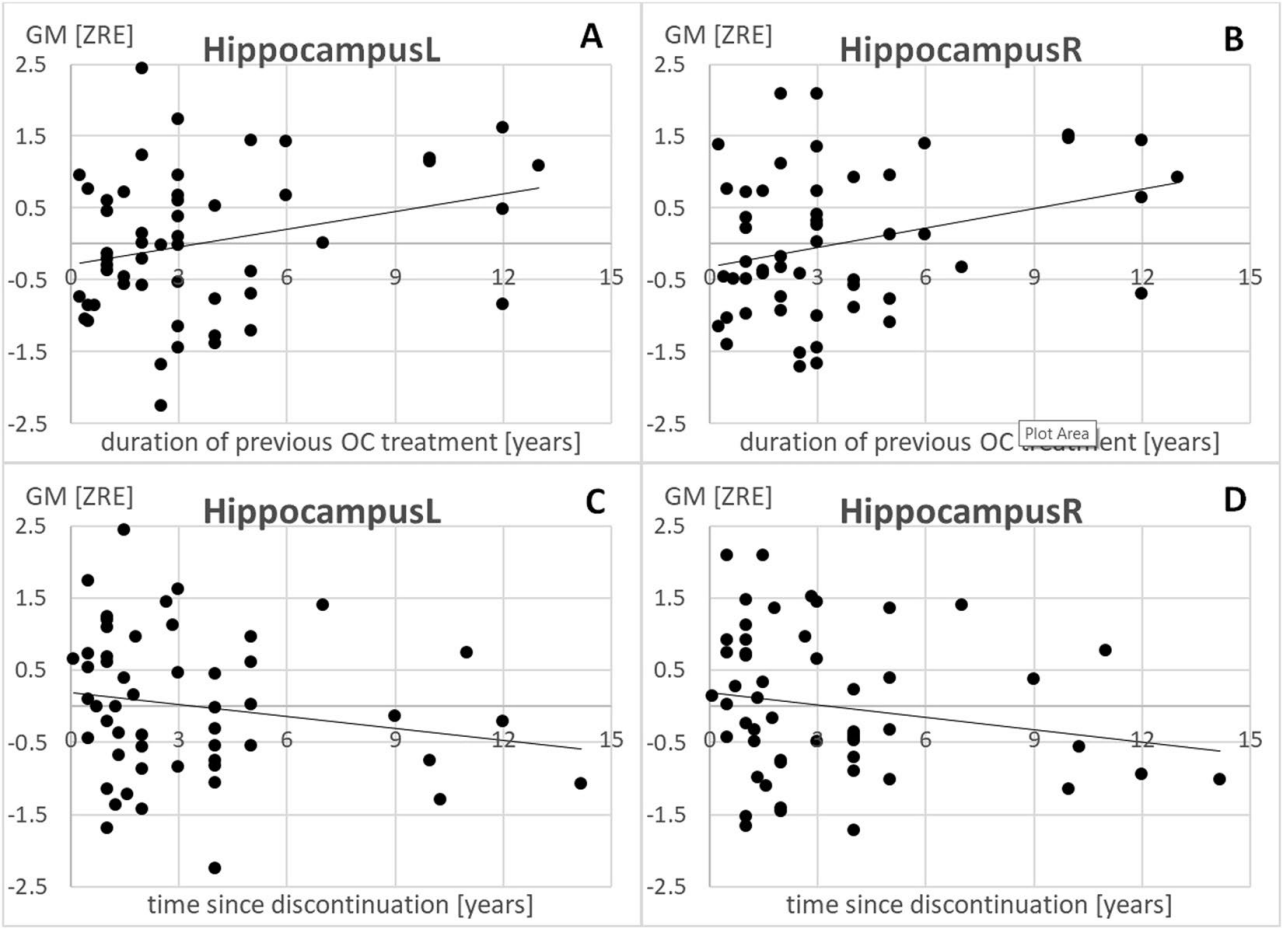
Relationship of previous OC treatment duration and time since discontinuation to gray matter volumes in the left and right hippocampus. L = left, R = right, ZRE = standardized residual after controlling for age and total intracranial volume.

As hypothesized there was a significant positive association between duration of previous OC treatment and hippocampal volumes bilaterally (left: r = 0.29, p_FDR_ = 0.02; right: r = 0.31, p_FDR_ = 0.02; Fig. 1A,B). The longer participants had previously used OCs, the larger was their hippocampus bilaterally. There was a significant negative association between time since discontinuation and grey matter volumes of the hippocampi bilaterally (left: r = −0.27, p_FDR_ = 0.045; right: r = −0.27, p_FDR_ = 0.045; Fig. 1C,D). Furthermore, the effect of previous OC treatment duration did not survive, when controlling for time since discontinuation in the model (left: r = 0.18, p_FDR_ = 0.09; right: r = 0.21, p_FDR_ = 0.09).

#### Basal ganglia

**Figure 2:**
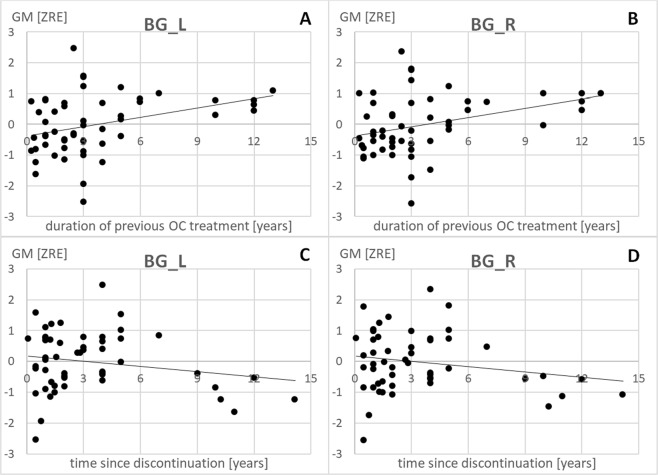
Relationship of previous OC treatment duration and time since discontinuation to gray matter volumes in the left and right basal ganglia (BG). L = left, R = right, ZRE = standardized residual after controlling for age and total intracranial volume.

Likewise, there was a significant positive association between duration of previous OC treatment and basal ganglia volumes bilaterally (left: r = 0.37, p_FDR_ = 0.01; right: r = 0.37, p_FDR_ = 0.01; compare Table 1, Fig. 2A,B). The longer participants had previously used OCs, the larger were their basal ganglia bilaterally. Again, there was a significant negative association between time since discontinuation and grey matter volumes of the basal ganglia bilaterally (left: r = −0.24, p_FDR_ = 0.045; right: r = −0.24, p_FDR_ = 0.045; Fig. 2C,D). However, for the basal ganglia, the effect of previous OC treatment survived, when controlling for time since discontinuation in the model (left: r = 0.29, p_FDR_ = 0.04; right: r = 0.29, p_FDR_ = 0.04).

#### Other areas

At the whole brain level, no additional effects of previous OC treatment or time since discontinuation in other brain areas were observed. Also the hippocampus and basal ganglia did not survive the whole-brain level corrected significant threshold.

### Effect of androgenicity

Among the 52 women, who had previously used only one OC for a continuous time period, 14 reported use of an anti-androgenic OC, 18 reported use of an androgenic pill and 20 did not recall the brand of OC they had previously used. When including androgenicity in the regression models reported above, there was no significant effect of androgenicity on hippocampal grey matter volumes and androgenicity did not interact with the duration of previous OC treatment or the time since discontinuation of OC treatment (all |β| < 0.29, all |t| <1.41, all p > 0.16). Separate partial correlation analyses controlling for age and TIV in previous users of androgenic and anti-androgenic OC revealed the following.

For the hippocampus, positive associations to the duration of previous OC-treatment were observed for both androgenic OC (left: r = 0.27, p = 0.15; right: r = 0.29, p = 0.14) and anti-androgenic OC (left: r = 0.53, p = 0.04; right: r = 0.61, p = 0.02), albeit effects were only significant in users of anti-androgenic OC. A non-significant negative association to time since discontinuation was also observed in both androgenic OC (left: r = −0.43, p = 0.05, right: r = −0.37, p = 0.08) and anti-androgenic OC (left: r = −0.32, p = 0.15; right: r = −0.34, p = 0.14).

Likewise, for the basal ganglia, positive associations to the duration of previous OC-treatment were observed for both androgenic OC (left: r = 0.42, p = 0.05; right: r = 0.44, p < 0.05) and anti-androgenic OC (left: r = 0.54, p = 0.04; right: r = 0.60, p = 0.02). For androgenic OC, time since discontinuation was significantly negatively related to BG volumes (left: r = −0.43, p < 0.05; right: r = −0.43, p < 0.05), while for anti-androgenic OC, time since discontinuation was not related to BG volumes (left: r = 0.07, p = 0.41; right: r = −0.21, p = 0.26).

## Discussion

The present study aimed to investigate the effect of previous OC treatment on gray matter volumes in the hippocampus and basal ganglia. Endogenous estradiol has been shown to relate to increased volumes of the hippocampus, while endogenous progesterone has been shown to relate to increased volumes of the basal ganglia^12,13^. Since OC contain both estrogenic and progestogenic components, we hypothesized that – in women who are not current users of OC – the duration of previous OC treatment would relate positively to gray matter volumes in the hippocampus and basal ganglia. Furthermore, we hypothesized that the time since discontinuation of OC treatment would relate negatively to gray matter volumes in the hippocampus and the basal ganglia and that controlling for the time since discontinuation would alleviate the effect of previous treatment duration.

Our results demonstrate that the duration of previous OC treatment was indeed positively related to gray matter volumes in the hippocampus and the basal ganglia, while the time since discontinuation was indeed negatively related to gray matter volumes in the hippocampus and the basal ganglia. These results are in line with previous studies demonstrating larger hippocampal and basal ganglia volumes in OC users compared to non-users^17^, but see^23^. However, controlling for time since discontinuation reversed the effects of previous treatment duration only in the hippocampus, but not in the basal ganglia. We assume that these results are attributable to the fact, that effects of previous OC treatment on the hippocampus, while detectable after discontinuation, reverse in the same time frame as they emerged. This may also explain why no differences emerged between women who had previously used OCs and women who had never used OCs. However, in the basal ganglia, reversibility of effects may take longer than actual treatment duration. The fact that particularly the BG display effects of previous OC treatment duration that are not fully reversed by the time since discontinuation, are especially interesting in light of findings that previous OC treatment predicts later diagnosis of depression^24^, prescription of anti-depressants^25^ and psychotropic drug use^26^. All these effects appear to be particularly severe if OC are prescribed during adolescence.

Regarding the effects of endogenous progesterone on the basal ganglia, we previously discussed that the reduction in right basal ganglia volume after the mid-luteal progesterone peak might mediate premenstrual mood symptoms^13^. This interpretation was due to the fact that mood disorders like unipolar and bipolar depression^27^ have been linked to a reduction in basal ganglia volumes. Likewise, smaller basal ganglia volumes have been linked to depressive symptoms in neurological disorders like Parkinsons disease^28^. Accordingly, a later diagnosis of depression in women who have previously used OC may not so much be attributable to the fact that OC treatment appears to increase basal ganglia volumes, but to the process of slowly reversing these effects after discontinuation of OC treatment.

As a limitation of the present study it has to be noted that effects of the androgenicity of the progestin component could only be explored but are not conclusive, since a majority of women was not able to recall, which brand of contraceptives they had previously used. The preliminary results on the matter suggest that in the structures investigated for this study, i.e. the hippocampus and the basal ganglia, effects of previous contraceptive treatment were observed irrespective of the androgenicity of the progestin component. This is plausible, since the effects were postulated based on estrogenic and progestogenic actions and not based on androgenic or anti-androgenic actions. However, for the basal ganglia it appears that time since discontinuation has a stronger effect in androgenic compared to anti-androgenic OC users. Accordingly, the failure to fully reverse the effect of previous treatment duration by the time since discontinuation may be attributable to the group of anti-androgenic OC users. This observation may be related to the fact that anti-androgenic progestins like Drospirenone have a longer half-life than androgenic progestins like levonorgestrel^29^.

Furthermore, it has to be pointed out that apart from age, other confounding variables related to previous OC-use, like social status or relationship status were not assessed in the present study. Particularly, the decision to start OC treatment is oftentimes related to entering a long-lasting relationship, while the decision to discontinue OC treatment is often related to a break up. Relationship dynamics may well play a role in women’s psychological well-being and future studies should focus on disentangling the effects of OC treatment from relationship status.

In summary, our results demonstrate that some effects of OC on brain structure, particularly in the hippocampus and the basal ganglia, may last well beyond the period of contraceptive treatment. These findings question the immediate reversibility of OC effects on brain structure.

## Methods

### Participants

The sample comprised 131 healthy naturally cycling women with a mean age of 24.21 years (SD = 4.01 years, range: 18–35 years), who participated in one of three functional imaging studies and provided information about their previous contraceptive use. Participants were recruited via flyers at the University of Salzburg and via Online advertisements. All three studies address the effects of endogenous sex hormones on brain structure or function and results have in part been published^13,14^. Information on previous contraceptive use is collected as part of our standard screening questionnaire regarding women’s hormonal status. All women had a regular menstrual cycle of 21–35 days (mean duration: 28.5 days, SD = 2.74 days).

Since all studies focused on the effects of sex hormones on the brain, the young age range was chosen to minimize confounds by (i) use of multiple different OCs, (ii) previous pregnancies, and (iii) age-related volume decline. With higher age, the likelihood of a woman having tried multiple contraceptive methods or having had multiple previous pregnancies increases. In addition, a more homogenous sample with regards to age minimizes confounds due to age-related decline in grey matter volumes. On the one hand, the areas of interest in the present study are subject to age related decline^30^. On the other hand, also total intracranial volume, which is an important covariate in studies on regional grey matter volumes, declines with age and the rate of the age related decline may differ between regions^31^. Accordingly, an age homogenous sample is less likely to involve confounds due to age effects on brain volumes. Furthermore, the duration of previous contraceptive use is of course limited by a woman’s age, such that a younger age-homogenous sample minimizes the collinearity between those variables.

Since hippocampal volume is also affected by the natural menstrual cycle^13^, scanning sessions were scheduled in the early follicular phase of the menstrual cycle (cycle days 1–8) for the majority of women (109 women). Unfortunately, 22 women were unavailable during their early follicular phase. Their scanning sessions were thus scheduled during the mid-luteal phase (3–10 days before onset of next menses), since hippocampal volume did not differ between early follicular and mid-luteal cycle phase in our previous study^13^.

Of the 131 women, 52 had never used any hormonal contraceptives, while 79 reported previous use of at least one oral hormonal contraceptive (OC). Of those 80 women, all were able to provide the total duration of previous OC use in years and the month in which they had stopped using the last OC. 52 had used only one OC before for a continuous time period, and 27 had used multiple different contraceptive methods (including IUD, NuvaRing, patches). Since the respective influence of various contraceptive methods is hard to disentangle, we focus our analysis on the 52 women, who had used only one OC before. On average, they had used an OC for 3.60 years (SD = 3.31, range: 0.25–13 years) and had discontinued OC treatment 3.32 years ago (SD = 3.27 years, range: 0.08–15 years). Among those 52 women, only six were scanned during their luteal cycle phase – the rest were scanned during menses. To address potential effect of cycle phase, all analyses (see below) were rerun, after excluding women who were scanned in their luteal cycle phase. Since the results did not change, we report the results of the whole sample of 52 women.

Unfortunately, 20 women were not able to recall the brand names of all OC they had previously used. Among the remaining women, the progestins contained in the OC they had been using were classified as androgenic in 18 women (Levonorgestrel, Desogestrel, Dienogest, etc.) and anti-androgenic in 14 women (Drospirenone, Chlormadinonacetat, etc.).

### Ethics statement

The studies, during which the data described in this manuscript were acquired, were all approved by the University of Salzburg’s ethics committee. All methods conform to the Code of Ethics World Medical Association (Declaration of Helsinki). All subjects gave their informed written consent to participate in the studies.

### MRI data acquisition

High resolution structural images were acquired on a Siemens Magnetom TIM Trio 3 Tesla scanner (Siemens Healthcare). A T1-weighted 3D MPRAGE sequence (160 sagital slices, slice thickness = 1 mm, TE 291 ms, TR 2300 ms, TI delay 900 ms, FA 9°, FOV 256 × 256 mm) was utilized. In all studies, the MPRAGE was acquired as fourth scan after a fieldmap, a resting state functional scan, and a 20–30 min task-based functional scan with tasks varying between studies.

### MRI data analysis

Data were segmented into gray and white matter partitions using the CAT12 toolbox (http://dbm.neuro.uni-jena.de/vbm/) as implemented in the SPM12 software (http://www.fil.ion.ucl.ac.uk/spm/). We used SPM12 tissue probability maps and European brain templates for affine regularization during the initial SPM12 affine registration, as well as light affine preprocessing and moderate (0.5) strength of local adaptive segmentation, skull stripping and final clean-up. The different brain segments were modulated using nonlinear normalization parameters to control for individual differences in brain size. In a first step, gray matter volumes from the left and right hippocampus were extracted using the get_totals script by by G. Ridgeway (http://www0.cs.ucl.ac.uk/staff/gridgway/vbm/get_totals.m). Aal-masks for the hippocampus and basal ganglia (putamen/pallidum) were created using the wfu-pickatlas. These are the same masks as used in our previous study on the effects of endogenous hormones^13^.

In a second step, whole-brain regressions were performed using SPM12 second level analyses, to explore, whether previous contraceptive use affected gray matter volumes in other areas besides the hippocampus or basal ganglia. To that end, gray matter segments were smoothed using an 8 mm full width at half maximum (FWHM) Gaussian kernel. Following an uncorrected primary threshold of p < 0.001, a secondary cluster-level FDR-corrected threshold of p < 0.05 was used.

### Statistical analyses

Statistical analyses were performed using partial correlation analyses and multiple regression models in SPSS Statistics 24 software. Group comparisons were performed using independent samples t-tests. Correlation and regression analyses focus on the 52 women, who had used only one OC before. Since it was not possible to control beforehand, how many participants of our studies had previously used one OC, post-hoc power-analyses were performed using G*Power software. For a sample of 52, a power of 85% was estimated to detect effects of r = 0.30 in linear regression analyses.

Dependent variables were grey matter volumes of the left and right hippocampus, as well as the left and right basal ganglia. Independent variables were duration of previous OC treatment, as well as the time since discontinuation of OC treatment. All analyses controlled for age and TIV. Since we had specific hypotheses regarding the direction of effects, one-sided significance tests were performed and p-values were FDR-corrected for multiple comparisons across the four regions of interest (p_FDR_).

In a first step we addressed whether duration of previous OC treatment related positively to hippocampal and basal ganglia volumes using partial correlations controlling for age and TIV. In a second step we addressed whether the time since discontinuation of OC treatment related negatively to hippocampal and basal ganglia volumes using partial correlations controlling for age and TIV. In a third step we addressed, whether the effect of previous treatment duration was reversed by the time since discontinuation using partial correlations controlling for age, TIV and time since discontinuation. To address potential influences of menstrual cycle phase, all analyses were rerun after excluding women who were scanned in their luteal cycle phase, which did not significantly affect the results.

To explore potential influences of the androgenicity of the previously used OCs, linear regression models were run including age and TIV as covariates as no interest, as well as duration of previous OC treatment/time since discontinuation, androgenicity and their interaction as effects of interest. Since many women were unable to recall the brand of OC they had used, for these preliminary multiple regression analyses, androgenicity was coded as follows (0 = androgenicity unknown, 1 = androgenic, −1 = anti-androgenic). In addition, we performed the partial correlation analyses described above separately for women, who reported use of an androgenic OC and women, who reported use of an anti-androgenic OC, but excluding women who didn’t recall the OC they had used. Since these analyses include only small samples, results need to be considered as preliminary.

Due to the homogenous age range chosen, the duration of previous OC-use was not significantly related to age (r = 0.25, p = 0.07) and also independent of the time since discontinuation (r = −0.19, p = 0.19). This reduced collinearity among variables is favorable for the partial correlations and multiple regression models performed in the present study.

### One sentence summary

In women, who are not current users of hormonal contraceptives, the duration of previous contraceptive use correlates positively with hippocampal and basal ganglia volumes.

## Data Availability

Data are openly available at http://webapps.ccns.sbg.ac.at/OpenData/.
